# Growth performance, blood composition, carcass characteristics, and meat quality of Ossimi and Barki fattening lambs as influenced by breed type and zilpaterol hydrochloride supplementation

**DOI:** 10.1093/tas/txag059

**Published:** 2026-05-09

**Authors:** Ahmed M A Hussein, Mahmoud M Abdelsattar, Einar Vargas-Bello-Pérez, Yanliang Bi

**Affiliations:** Animal Production Departement, Faculty of Agriculture, Assuit University, Assuit 71515, Egypt; Institute of Feed Research of Chinese Academy of Agricultural Sciences, Key Laboratory of Feed Biotechnology of the Ministry of Agriculture and Rural Affairs, Beijing 100193, China; Department of Animal and Poultry Production, Faculty of Agriculture, Qena University, Qena 83523, Egypt; Department of International Development, School of Agriculture, Policy and Development, University of Reading, Earley Gate, Reading RG6 6EU, United Kingdom; Facultad de Zootecnia y Ecología, Universidad Autónoma de Chihuahua, Periférico R. Aldama Km 1, Chihuahua 31031, México; Institute of Feed Research of Chinese Academy of Agricultural Sciences, Key Laboratory of Feed Biotechnology of the Ministry of Agriculture and Rural Affairs, Beijing 100193, China

**Keywords:** zilpaterol hydrochloride, carcass, meat quality, fattening lambs, Egyptian local breeds

## Abstract

To study the effect of zilpaterol hydrochloride (ZH), 60 lambs were divided into six groups of different breeds (Ossimi or Barki) receiving 0 [CON], 0.10 [Z1], or 0.15 [Z2] mg/kg BW. Ossimi had higher feed intake, carcass traits, blood hemoglobin, and neutrophils, but lower meat protein and blood basophils. Dietary ZH was associated with linear improvements in feed intake, feed conversion ratio, body weight, daily gain, meat protein, pH, tenderness, and water holding capacity, blood hemoglobin, hematocrit, red blood cells, neutrophils, glucose, total protein, insulin-like growth factor 1, triiodothyronine, and thyroxine levels. In contrast, dietary ZH was associated with linear decreases in meat fat and in plasma cholesterol and triglyceride levels. The weights of the kidney fat, supraspinatus muscle, flank, best rib, and best rib fat and bone proportions were quadratically influenced by dietary ZH. Breed by diet interactions were observed, with Z2 × Barki showing increased values for carcass weight, dressing percentage, liver, right side, left side, shoulder, round, ribs, and longissimus, supraspinatus, and semimembranosus muscles compared to CON. Conversely, Z2 × Ossimi exhibited decreased values for these traits. Notably, ZH treatment reduced kidney and omental fat in Barki, and increased these in Ossimi.

## Teaser

ZH supplementation was associated with improved growth, meat quality, hematological and immune parameters, and anabolic hormone levels, concurrent with reduced plasma lipids and meat fat. Barki lambs demonstrated greater improvements in carcass traits, suggesting breed-specific responsiveness.

## Introduction

Sheep production is becoming increasingly important in Egypt, as it addresses animal protein deficiencies in human diets (Fereig et al. 2023). In Egypt, sheep are primarily reared for meat and lamb production, with wool and milk as the secondary products. Barki and Ossimi sheep, two indigenous Egyptian breeds, are valued for their adaptability to Egypt’s hot and dry conditions and limited feed resources (Aboulnaga and Abdelsabour 2023). However, there were notable variations between Barki and Ossimi lambs. Barki lambs are known to produce high-quality meat under harsh desert conditions by utilizing extensive transhumance grazing techniques in the Mediterranean coastal zone (Abousoliman et al. 2020; Fereig et al. 2023). In contrast, Ossimi lambs were raised in more intensive systems in the Nile Valley and Delta. They are recognized for their superior performance and body growth compared to those of Barki lambs (Aboulnaga and Abdelsabour 2023). In addition, although Barki and Ossimi lambs are both fat-tailed breeds, they have a markedly lower fat tail than Ossimi lambs (El-Malky et al. 2019). Therefore, the Barki and Ossimi breeds were compared in this study due to their differences in growth performance, meat quality, environmental adaptability, and genetic diversity. By comparing these breeds, we can better understand their potential and role in Egyptian sheep production, and how to improve their production efficiency and meat quality through feeding management.

The use of exogenous growth-enhancing molecules, such as β-adrenergic agonists (βAs), to improve production efficiency is prohibited by the European Union (EU) owing to regulatory and safety concerns (Niño et al. 2017). However, securing access to export markets for these drugs is challenging (Smythe 2024). Therefore, a mechanistic understanding of how these growth promoters function across different sheep breeds, feeding regimens, and management systems is essential for optimizing their application while maintaining compliance with international standards. Zilpaterol Hydrochloride (ZH), a prominent feed additive, has been approved for use in beef feedlots in various countries, including South Africa, Mexico, and the USA, and has been used in the USA since 2006 (Webb et al. 2024). The primary effect of ZH on ruminant production is linked to the enhancement of protein synthesis, inhibition of lipogenesis, and promotion of muscle hypertrophy in livestock by binding to β-adrenergic receptors (Johnson et al. 2014; Lopez-Baca et al. 2019). Therefore, ZH has shown promising effects on growth performance, energy utilization efficiency, and carcass traits in lambs when administered appropriately during the final fattening phase (Lopez-Baca et al. 2019; Robles et al. 2024). Similarly, in beef finishing diets, ZH enhanced average daily gain (ADG), feed conversion ratio (FCR), and carcass traits by improving skeletal muscle growth and lean tissue development (Johnson et al. 2014). The growth-promoting effect of ZH primarily depends on the presence and quantity of type II muscle fibers (Vahedi et al. 2015). Recently, Motsinger and Gonzalez (2024) reported that the increase in myofibrillar protein synthesis by ß-adrenergic agonists is caused by increased cyclic adenosine monophosphate (cAMP) activity, which may activate mammalian target of rapamycin (mTOR). However, its impact on protein degradation and intramuscular adipose tissue has not yet been established. They suggested that depression in the marbling score could be due to the dilution of marbling over a greater longissimus cross-sectional area. However, there are potential effects on meat quality, including reductions in tenderness (Fulton et al. 2024) and alterations in color parameters (Cayetano-De-Jesus et al. 2020; Guerrero-Bárcena et al. 2023).

Differences in muscle and adipose composition in Egyptian local breeds could result in different responses to ZH. This underscores the critical need to investigate the effect of ZH supplementation across different doses and sheep breeds. Blood composition is a crucial indicator of metabolic status (Lynch et al. 2010). Alterations in blood parameters can reveal the metabolic pathways affected by ZH and the potential breed-specific responses (Vicente Pérez et al. 2020; Abdelsattar et al. 2022). Furthermore, hematological parameters provide insights into potential stress responses or physiological adaptations to β-adrenergic stimulation (Reith et al. 2022; Howell et al. 2024). Therefore, this study aimed to determine the effects of ZH on growth performance, blood composition, carcass attributes, and meat quality in fattening Barki and Ossimi lambs, the dominant Egyptian breeds. We hypothesized that the response to ZH as a strategy to decrease body fat reserves and increase lean gain would differ between Ossimi and Barki lambs. Understanding the influence of ZH on different breeds could contribute to optimizing livestock production practices and enhancing the economic viability of sheep farming in Egypt.

## Materials and methods

### Animals, management, and feeding

The Animal Care and Ethics Committee of the Animal Production Department, Faculty of Agriculture, Assuit University, approved all animal procedures (APCC-2017-57/04). Sixty healthy growing Egyptian local lambs at EL-Hamd farm, Sohag, Egypt, were meticulously selected from two pure breeds, Barki (*n* = 30) and Ossimi (*n* = 30). At the beginning of the study, lambs were 10 mo old and had an average body weight (BW) of 32.7 ± 1.38 kg for Barki lambs and 41.0 ± 1.44 kg for Ossimi lambs. Similar BW variations were observed by Ashour et al. (2020). They emphasized that Ossimi lambs weighed 44.93 ± 1.82 kg and Barki lambs weighed 35.90 ± 1.30 kg at 10 to 12 mo.

Before the start of the study, all lambs received external treatment with albendazole and levamisole. Furthermore, they received vaccination against foot and mouth disease (FMD) within 2 d of their selection. The lambs were categorized into two breed-based groups. Within each breed-based group, lambs were subdivided into three treatments, each comprising ten. The lambs were reared individually in separate pens (180 × 90 cm) with a concrete floor equipped with water and feed bunks.

Sixty male lambs were randomly assigned into six groups of 10 lambs within each group, representing two breeds and three dietary treatments, as follows: Ossimi lambs offered a basal diet without any additional β-agonists supplements (CON), Ossimi lambs receiving a low dose of ZH (Zilmax, Intervet Inc., Millsboro, DE, USA) at a rate of 0.10 mg/kg^−1^ BW per lamb (Z1), and Ossimi lambs receiving a high dose of ZH at a rate of 0.15 mg/kg^−1^ BW per lamb (Z2), as well Barki lambs without ZH, Barki lambs with a low ZH dose, and Barki lambs with a high ZH dose. Randomization was performed using a random number generator (Excel, Microsoft Corp., Redmond, WA, USA). Even though the dosage of ZH for goats has not yet been established, maximal responses have been observed at 20 mg ZH kg live weight (Estrada-Angulo et al. 2008; López-Carlos et al. 2014). This was the rationale for the concentration used in the present study. All lambs across the study were provided with commercial mixed concentrate feed and chopped wheat straw at a concentrate: roughage ratio of 80:20. The diets were formulated to achieve 250 g/d of ADG, considering the requirements for late maturity, according to NRC (2007). Each morning prior to the 0800 h meal, the feed bunks were inspected 30 min beforehand to evaluate the previous day’s intake. Any leftover feed was cleared, and these data were used to adjust the afternoon feed allocation, maintaining a refusal rate below 10%. The ingredients and chemical composition of the concentrate and chopped wheat straw are listed in Table 1.

**Table 1:** Feed composition and nutrient content of the concentrate mixture and wheat straw.

Items	Concentrate	Wheat straw
**Ingredients (%)**		
** Corn**	42.0	–
** Wheat bran**	30.0	–
** Corticated cotton meal**	25.0	–
** Trace mineral and vitamin premix^a^**	1.50	–
** Sodium chloride**	1.50	–
**Chemical composition (% on DM basis)**		
** Crude protein**	13.7	3.11
** Crude fiber**	13.9	27.2
** Ether extract**	3.58	2.87
** Nitrogen free extract**	59.2	53.2
** Ash**	9.74	13.7
** Total energy^b^ (Mcal/kg DM)**	4.14	3.78

a The premix consist of (per kg): 20,000,000 IU vitamin A, 10,000 mg vitamin E, vitamin 200,000 IU D3, 10,000 mg Fe, 2500 mg Cu, 100 mg Mo, 20,000 mg Mn, 100 mg Co, 800 mg I, 20,000 mg Zn and 100 mg Se.

b Gross energy (GE) was calculated according to NRC (2007).

Fresh water was provided ad libitum. According to the method described by Dickerson et al. (2022), β-agonist additives were blended with 200 g of ground corn and administered individually at 8 am daily, with the quantity adjusted according to the lambs’ BW. In the CON group, lambs received 200 g of ground corn at 8 am without any additives. All personnel involved in carcass dissection, meat quality analysis, and blood parameter measurements were blinded to treatment assignments. Samples were identified only by numerical codes, and treatment codes were revealed only after all analyses were completed.

Energy intake was calculated by multiplying dry matter intake (DMI) by the diet’s average gross energy (GE) content for the entire experiment. Following BW measurements, the β-agonist additive quantities were recalculated based on the new BW. The administration of β-agonists was continued for 30 d. Individual BW was recorded before the morning feeding on days 0, 10, 20, and 30 of the experiment. The ADG was calculated for the overall period as the difference between the final and initial BW and the total weight gain, and divided by the number of feeding days. In addition, FCR was calculated as the ratio of feed intake to weight gain.

### Blood sampling and analysis

Blood samples were obtained at 0700 h after an 8-h fasting period. Sampling was performed on 30 d of the experiment. Blood samples were extracted from the jugular vein utilizing EDTA vacutainer tubes (approximately 6 mL) and heparinized syringes (4 mL). The 4 mL tubes were used to determine the complete blood count (CBC). The remaining tubes (6 mL) were centrifuged at 4,000 × *g* for 20 min to obtain plasma samples, which were preserved at −20°C for subsequent analysis. In the analyses, the complete blood cell count, encompassing parameters such as hemoglobin (HB) concentration, red blood cell count (RBCs; million cells/µL), hematocrit (HCT; %), mean corpuscular volume (MCV; fL), mean corpuscular hemoglobin (MCH; pg), mean corpuscular hemoglobin concentration (MCHC; g/dL), platelet count (1000 cells/µL), as well as white blood cell count (1000 cells/µL) (WBC) and its differential count (neutrophils, lymphocytes, monocytes, eosinophils, and basophils; %), were determined using a semi-automatic biochemical blood analyzer (model Emperor EMP-168) according to the manufacturer’s specifications. Hematological analysis of blood samples was conducted at the Veterinary Hematology Laboratory, Faculty of Veterinary Science, Assiut University, Egypt.

Plasma parameters were analyzed at the Central Laboratory of the Faculty of Agriculture, Assuit University, Egypt. Plasma concentrations of total protein (TP), alanine aminotransferase (ALT), aspartate aminotransferase (AST), creatinine, cholesterol, triglycerides, glucose, and urea nitrogen (BUN) were measured using commercial colorimetric assay kits (Spectrum Diagnostics, Egypt). Absorbance was monitored using a spectrophotometer (model PG Instruments Limited T80) set at 546 nm for all measurements, except BUN, which was measured at a wavelength of 578 nm (Reda 2018). Plasma triiodothyronine (T3), thyroxin (T4), and cortisol levels were determined using a plate reader with a kit from Novus Biologicals, following the manufacturer’s instructions. Plasma insulin-like growth factor 1 (IGF1) was determined using an ELISA kit specific for sheep (Assay Reagent Genie, Dublin, Ireland; #SHFI00042; sensitivity, 0.563 ng/mL) following the manufacturer’s protocol and a plate reader. The assay employed a biotin-conjugated antibody and Horseradish Peroxidase (HRP)-streptavidin for detection.

### Slaughter and carcass measurements

Upon completion of the 30-d experimental period, lambs were slaughtered after a 12-h fast, during which they had unrestricted access to water. The weights of non-carcass components, such as the liver, were promptly documented. Subsequently, the tail, caul, perirenal, and omental fat were meticulously isolated and quantified. Subsequently, the weight of the carcass that retained its elevated temperature was determined. Additionally, the dressing percentage was calculated as the ratio of hot carcass weight to live BW. The carcass was divided along its midline into right and left halves, which were individually weighed. Subsequently, the left half was subdivided into seven distinct cuts: the loin, shoulder, ribs, brisket, round, neck, and flank. The section encompassing the 9^th^ to 11^th^ ribs from the left side was isolated, weighed, and subsequently disassembled into its constituent parts: muscle, fat, and bone. The semimembranosus, longissimus, and supraspinatus muscles were meticulously dissected and weighed. The longissimus muscle was used to assess meat quality parameters and was frozen at −20°C.

### Meat quality

The meat of the lambs was analyzed for chemical composition, including moisture, protein, and fat, according to AOAC (2005) methods. In addition, muscle pH was measured 24 h postmortem as described by Gökalp et al. (2001). Approximately 10 g of the meat sample, maintained at 4°C, was finely diced and homogenized in 100 mL of distilled water. The mixture was filtered, and the pH was measured using a digital pH meter (Model AD1030). The electrodes were meticulously cleaned with distilled water and dried with filter paper before each use and between analyses of different samples. Subsequently, the spear electrode was inserted into the sample, and pH meter readings were recorded. Furthermore, approximately 300 ± 0.1 mg was extracted from each muscle sample to assess water-holding capacity using the compression method described by Sami et al. (2012). Each meat specimen was placed on filter paper (Whatman No. 0.1) and then sandwiched between two glass sheets. A 1 kg weight was applied to the upper glass sheet for 10 min to ensure sufficient pressure. Subsequently, the samples were reweighed, and the water-holding capacity (WHC) was quantified as the percentage of loss relative to the initial weight. Tenderness of the cooked meat samples was measured by determining the shear force. The meat samples were thawed overnight at 4°C and sliced into 20 mm thick chops. Each lamb chop was then cooked in a continuously boiling water bath until a final core temperature of 75°C was reached, followed by immediate immersion in ice water for equilibration. After cooling, six cores with a diameter of 1.27 cm, devoid of fat or connective tissue and aligned parallel to the longitudinal orientation of the muscle fibers, were extracted from each lamb chop. The Warner-Bratzler shear force (WBS) was determined using an Instron Universal Testing Machine (Model Series IX; Instron Co., Norwood, MA, USA) equipped with a Warner-Bratzler shearing device. The samples were sheared perpendicular to the core’s longitudinal axis, and the WBS was defined as the peak force observed on the curve (Honikel 1998).

### Statistical analysis

Prior to the experiment, G*Power 3.1 software (Heinrich-Heine-Universität Düsseldorf, Düsseldorf, Germany) was used to determine the required sample size per treatment group (Hintze 2008). The primary response variable was BW. For the main effect of breed, based on data from Ashour et al. (2020) showing differences in BW (Ossimi: 44.93 ± 4.815 kg; Barki: 35.90 ± 5.035 kg; Cohen’s d = 1.833), a sample of 6 lambs per breed would provide 80% power at α = .05 using a two-sample t-test. However, because the study employed a 2 × 3 factorial design requiring detection of breed × treatment interactions, sample size was calculated based on the expected interaction effect. Assuming the interaction would explain approximately 15% of the variance (equivalent to Cohen’s f = 0.42), with α = .05, power = 0.80, numerator df = 2, and 6 groups, the required total sample size was calculated as 58 lambs (≈10 lambs per group) using the 'ANOVA: Fixed effects, special, main effects and interactions’ procedure. This sample size ensures adequate power for both main effects and the interaction of interest.

All data were analyzed using the PROC MIXED procedure of SAS (SAS 2013) to assess the main effects of breed (Barki vs. Ossimi) and ZH level (0, 10, and 15 mg/kg BW), and their interaction as fixed effects. The initial BW was used as a covariate in analyses of BW-related data. BW, DMI, and GE intake were analyzed as repeated measurements. When the fixed effects or their interactions were significant (*P* < 0.05), treatment means were separated using the Tukey-Kramer multiple comparison test. The impact of the ZH levels was further tested using polynomial (linear and quadratic) contrasts.

## Results

### Productive performance

The growth parameters of lambs are listed in Table 2. The breed type × diet interaction did not affect any feedlot performance measured in this study (*P* > 0.05), except for BW, FCR, and GE intake (*P* < 0.01). Supplementation with ZH linearly increased DMI, resulting in a significant increase in GE intake (*P* < 0.01). In addition, ZH supplementation linearly enhanced (*P* < 0.01) lamb BW, total gain, ADG, and FCR compared with the CON treatment. The DMI and GE intake were higher in Ossimi lambs (*P* < 0.01) than in Barki lambs. However, breed type did not affect the other growth parameters of lambs (*P* > 0.05).

**Table 2:** Effect of breed type (B) and zilpaterol hydrochloride (ZH) treatment (T) and their interaction (B × T) on the growth performance of finishing lambs.

Items	Barki			Ossimi			*p* values				Contrast^b^	
	CON^a^	Z1	Z2	CON	Z1	Z2	B	T	B × T	time	L	Q
**BW, kg^1^**	40.0^c^ ± 0.07	41.6^b^ ± 0.08	42.0^a^ ± 0.08	40.2^c^ ± 0.08	41.2^b^ ± 0.08	42.3^a^ ± 0.07	ns	**	**	**	**	ns
**TG, kg**	4.56^f^ ± 0.10	6.04^d^ ± 0.10	7.28^b^ ± 0.08	5.52^e^ ± 0.11	6.90^c^ ± 0.13	8.3^a^ ± 0.16	ns	**	ns	–	**	ns
**ADG 0–30 d, g/d**	152.0^f^ ± 3.34	201.3^d^ ± 3.27	242.6^b^ ± 2.77	184.0^e^ ± 3.59	230.1^c^ ± 4.49	277.2^a^ ± 5.41	ns	**	ns	–	**	ns
**DMI, kg/d**	1.19^b^ ± 0.03	1.43^a^^b^ ± 0.04	1.54^a^^b^ ± 0.04	1.53^a^^b^ ± 0.04	1.55^a^^b^ ± 0.03	1.70^a^ ± 0.02	*	**	ns	**	**	ns
**FCR**	7.87^a^ ± 0.18	7.12^b^ ± 0.12	5.90^c^ ± 0.06	8.35^a^ ± 0.17	6.76^b^ ± 0.14	6.16^c^ ± 0.12	ns	**	**	–	**	ns
**GE intake, Mcal/d**	4.86^c^ ± 0.13	5.81^b^ ± 0.17	6.25^b^ ± 0.18	6.20^b^ ± 0.15	6.29^b^ ± 0.11	6.90^a^ ± 0.09	**	**	**	**	**	ns

*Note*: Means within the same row with different superscripts (^a–f^) are significantly different (*, *P* < 0.05; **, *P* < 0.01). Each value is expressed as mean ± standard error of the mean.

1 Data are covariate-adjusted LSM.

Abbreviations: BW, body weight; TG, total gain; ADG, average daily gain; DMI, dry matter intake; FCR; feed conversion ratio.

a Treatments: CON, control group; Z1, 0.10 mg/kg BW of ZH; Z2, 0.15 mg/kg BW of ZH.

b *p* values reported for the linear and quadratic contrasts of treatments.

### Carcass traits

The carcass traits of lambs are listed in Table 3. The results indicated that non-carcass components (liver, kidney fat, and tail fat), and commercial meat cuts (shoulder, brisket, flank, neck, lion, best rib, best rib meat, best rib bone, and best rib fat), and supraspinatus muscle were influenced by breed type (*P* < 0.01), with Ossimi Lambs being heavier than Barki lambs. However, breed type did not affect the hot carcass weight, dressing percentage, caul and omental fat, right side, left side, round, ribs, and semimembranosus and longissimus muscles of lambs during dietary supplementation (*P* > 0.05). Hot carcass weight, dressing percentage, non-carcass components (caul fat, omental fat, and tail fat), commercial meat cuts (right side, left side, shoulder, brisket, neck, lion, round, ribs, and best rib meat), and muscles (semimembranosus and longissimus) were not influenced (*P* > 0.05) by dietary supplementation with ZH. Liver weight was linearly (*P* < 0.05) influenced by ZH supplementation. In addition, the weights of kidney fat (*P* < 0.01), flank (*P* < 0.01), best rib (*P* < 0.01), best rib fat (*P* < 0.01), best rib bone (*P* < 0.01), and supraspinatus muscle (*P* < 0.05) were quadratically influenced by dietary ZH, and these parameters showed significant interaction between diet and breed type (*P* < 0.01) except of best rib bone (*P* > 0.05). A significant interaction between breed type and diet (*P* < 0.01) was also observed for the weight of carcass, dressing percentage, liver, right and left sides, shoulder, round, ribs, and the supraspinatus, semimembranosus, and longissimus muscles. Moreover, the breed by diet interaction was significant for best rib meat (*P* < 0.05). These values were highest in the CON × Ossimi group and lowest in the CON × Barki group. Moreover, the interaction of diet and breed type (*P* < 0.01) showed higher omental fat in Z2 × Ossimi lambs than in both CON and Z1 × Ossimi lambs. In contrast, the CON treatment of Barki lambs showed higher omental fat than Z2 treated barki lambs. No significant interactions between breed and diet were found for the values of caul fat, tail fat, brisket, neck, and lion.

**Table 3:** Effect of breed type (B) and zilpaterol hydrochloride (ZH) treatment (T) and their interaction (B × T) on carcass characteristics of finishing lambs.

	Barki			Ossimi			*p* values			Contrast^b^	
Items	CON^a^	Z1	Z2	CON	Z1	Z2	B	T	B × T	L	Q
**Hot carcass, kg**	19.0^f^ ± 0.33	20.4^e^ ± 0.36	21.9^d^ ± 0.47	28.1^a^ ± 0.54	26.3^b^ ± 0.47	24.8^c^ ± 0.40	ns	ns	**	ns	ns
**Dressing, %**	51.0^d^ ± 0.64	52.7 ^cd^ ± 0.61	54.6^b^^c^ ± 0.80	56.9^a^ ± 0.84	54.9^a^^b^ ± 0.64	53.1^cb^ ± 0.66	ns	ns	**	ns	ns
**Liver, g**	436.8^d^ ± 9.30	474.8 ^cd^ ± 10.2	500.2^c^ ± 10.6	733.9^a^ ± 30.4	692.9^a^ ± 35.9	577.5^b^ ± 11.9	**	**	**	**	ns
**Kidney fat, g**	305.7^b^ ± 14.7	205.2^d^ ± 10.3	199.9^d^ ± 7.26	256.5^c^ ± 8.70	264.5^c^ ± 13.4	397.7^a^ ± 19.4	**	**	**	ns	**
**Caul fat, g**	248.1 ± 97.6	164.4 ± 62.9	146.2 ± 57.6	188.9 ± 74.0	211.8 ± 80.4	325.2 ± 127.6	ns	ns	ns	ns	ns
**Omental fat, g**	422.6^a^^b^ ± 65.5	265.2^b^^c^ ± 39.9	238.6^c^ ± 37.1	305.7^b^^c^ ± 47.1	341.6^b^^c^ ± 51.3	549.7^a^ ± 85.3	ns	ns	**	ns	ns
**Tail fat, g**	2388^c^ ± 276.2	2149^c^ ± 252.6	1906^c^ ± 221.3	3769^b^ ± 438.3	4263^a^^b^ ± 501.6	5171^a^ ± 595.4	**	ns	ns	ns	ns
**Right side, kg**	9.52^f^ ± 0.17	10.3^e^ ± 0.20	11.0^d^ ± 0.25	14.1^a^ ± 0.29	13.2^b^ ± 0.26	12.4^c^ ± 0.20	ns	ns	**	ns	ns
**Left side, kg**	9.52^f^ ± 0.17	10.2^e^ ± 0.17	10.9^d^ ± 0.23	14.0^a^ ± 0.26	13.1^b^ ± 0.23	12.4^c^ ± 0.20	ns	ns	**	ns	ns
**Shoulder, kg**	1.84^d^ ± 0.04	2.05^c^ ± 0.04	2.12^c^ ± 0.07	2.72^a^ ± 0.08	2.64^a^ ± 0.05	2.40^b^ ± 0.05	**	ns	**	ns	ns
**Brisket, kg**	0.87^b^ ± 0.01	0.88^b^ ± 0.03	0.93^b^ ± 0.02	1.19^a^ ± 0.03	1.14^a^ ± 0.03	1.13^a^ ± 0.02	**	ns	ns	ns	ns
**Flank, kg**	0.26^c^ ± 0.01	0.24^c^ ± 0.01	0.32^b^ ± 0.01	0.41^a^ ± 0.01	0.31^b^ ± 0.01	0.33^b^ ± 0.01	**	**	**	ns	**
**Neck, kg**	1.04^b^ ± 0.03	1.11^b^ ± 0.04	1.13^b^ ± 0.05	1.46^a^ ± 0.06	1.43^a^ ± 0.04	1.35^a^ ± 0.03	**	ns	ns	ns	ns
**Lion, kg**	1.25^b^ ± 0.03	1.33^b^ ± 0.04	1.36^b^ ± 0.06	1.75^a^ ± 0.07	1.72^a^ ± 0.05	1.62^a^ ± 0.04	**	ns	ns	ns	ns
**Round, kg**	3.16^e^ ± 0.07	3.37^e^ ± 0.06	3.62^d^ ± 0.08	4.65^a^ ± 0.09	4.35^b^ ± 0.07	4.11^c^ ± 0.09	ns	ns	**	ns	ns
**Ribs, kg**	1.10^c^ ± 0.06	1.20^c^ ± 0.08	1.42^b^ ± 0.05	1.82^a^ ± 0.07	1.54^b^ ± 0.11	1.44^b^ ± 0.07	ns	ns	**	ns	ns
**Best rib, g**	393.9^c^ ± 17.4	366.2^c^ ± 12.0	488.9^b^ ± 23.8	628.0^a^ ± 30.9	471.9^b^ ± 15.6	512.1^b^ ± 21.8	**	**	**	ns	**
**Best rib meat, g**	239.1^c^ ± 9.8	245.2^c^ ± 9.3	270.5^c^ ± 11.8	347.3^a^ ± 15.0	315.9^a^^b^ ± 11.9	310.8^b^ ± 12.3	**	ns	*	ns	ns
**Best rib fat, g**	63.9 ^cd^ ± 9.92	43.6^d^ ± 5.75	118.8^b^ ± 13.6	152.7^a^ ± 17.7	56.2 ^cd^ ± 7.44	83.0^c^ ± 12.9	**	**	**	ns	**
**Best rib bone, g**	91.0^b^^c^ ± 4.90	77.4^c^ ± 4.36	99.6^b^ ± 7.04	128.0^a^ ± 9.15	99.8^b^ ± 5.63	118.3^a^ ± 6.19	**	**	ns	ns	**
**Supraspinatus, g**	114.6^e^ ± 2.64	141.8^d^ ± 3.13	157.8^c^ ± 3.49	202.5^a^ ± 4.08	182.7^b^ ± 4.09	149.0 ^cd^ ± 3.35	**	**	**	ns	*
**Semimembranosus, g**	102.1^d^ ± 1.78	121.9^c^ ± 2.85	151.5^b^ ± 5.91	194.5^a^ ± 7.46	157.1^b^ ± 3.75	132.8^c^ ± 2.20	ns	ns	**	ns	ns
**Longissimus, g**	67.2^d^ ± 1.55	94.3^c^ ± 2.80	117.0^b^ ± 3.76	150.2^a^ ± 4.80	121.5^b^ ± 3.67	87.4^c^ ± 1.96	ns	ns	**	ns	ns

*Note*: Means within the same row with different superscripts (^a–f^) are significantly different (*, *P* < 0.05; **, *P* < 0.01). Each value is expressed as mean ± standard error of the mean.

a Treatments: CON, control group; Z1, 0.10 mg/kg BW of ZH; Z2, 0.15 mg/kg BW of ZH.

b *p* values reported for the linear and quadratic contrasts of treatments.

### Meat quality

Table 4 shows the data obtained by analyzing the meat quality of Ossimi and Barki carcasses after the dietary supplementation experiment. Data were similarly unaffected by breed type (*P* > 0.05), except for the crude protein content, where Barki lambs showed higher values than Ossimi lambs (*P* < 0.05). In addition, the type of diet did not affect moisture, dry matter, or ash content (*P* > 0.05). However, crude protein content and pH increased linearly (*P* < 0.01) with ZH supplementation. In addition, water-holding capacity increased linearly (*P* < 0.01), with the CON group showing the lowest values. On the other hand, the fat percentage and Warner-Bratzler shear force decreased linearly (*P* < 0.01), with Z2 treatment showing the lowest values. Furthermore, no significant interactions between the breed and diet were observed (*P* > 0.05) for the meat quality parameters.

**Table 4:** Effect of breed type (B) and zilpaterol hydrochloride (ZH) treatment (T) and their interaction (B × T) on meat quality of finishing lambs.

Items	Barki			Ossimi			*p* values			Contrast^b^	
	CON^a^	Z1	Z2	CON	Z1	Z2	B	T	B × T	L	Q
**Moisture, %**	71.5 ± 0.13	71.3 ± 0.11	71.4 ± 0.13	71.5 ± 0.09	71.4 ± 0.28	71.4 ± 0.12	ns	ns	ns	ns	ns
**Dry matter, %**	28.5 ± 1.23	28.7 ± 1.11	28.6 ± 1.03	28.5 ± 0.92	28.6 ± 1.22	28.6 ± 1.67	ns	ns	ns	ns	ns
**Protein, %**	19.8^c^ ± 0.70	21.0^b^ ± 0.04	22.5^a^ ± 0.01	19.6c ± 0.13	21.0^b^ ± 0.01	22.3^a^ ± 0.04	*	**	ns	**	ns
**Fat, %**	7.74^a^ ± 0.11	6.72^b^ ± 0.14	5.22^c^ ± 0.06	7.98^a^ ± 0.02	6.73^b^ ± 0.14	5.41c ± 0.05	ns	**	ns	**	ns
**Ash, %**	0.96 ± 0.06	0.92 ± 0.20	0.91 ± 0.15	0.92 ± 0.12	0.90 ± 0.23	0.93 ± 0.12	ns	ns	ns	ns	ns
**pH**	5.80c ± 0.81	6.10^b^ ± 0.60	6.40^a^ ± 0.90	5.82^c^ ± 0.56	6.09^b^ ± 0.81	6.40^a^ ± 0.69	ns	**	ns	**	ns
**Water holding capacity, %**	72.0^b^ ± 2.31	76.0^a^ ± 2.61	76.0^a^ ± 3.72	72.0^b^ ± 4.01	76.0^a^ ± 3.44	76.0^a^ ± 2.92	ns	**	ns	**	**
**Warner–Bratzler shear force, kg**	4.20^a^ ± 0.33	3.60^b^ ± 0.12	2.20^c^ ± 0.10	4.20^a^ ± 0.14	3.60^b^ ± 0.35	2.20^c^ ± 0.12	ns	**	ns	**	**

*Note*: Means within the same row with different superscripts (^a–d^) are significantly different (*, *P* < 0.05; **, *P* < 0.01). Each value is expressed as mean ± standard error of the mean.

a Treatments: CON, control group; Z1, 0.10 mg/kg BW of ZH; Z2, 0.15 mg/kg BW of ZH.

b *p* values reported for the linear and quadratic contrasts of treatments.

### Blood hematology

Blood hematological data are shown in Table 5. There was no difference between treatments in platelet count, MCH, MCHC, MCV, WBC, lymphocytes, monocytes, and basophils (*P* > 0.05). However, the levels of HB, HCT, RBC, and neutrophils increased (*P* < 0.01) linearly with the supplementation of ZH. However, eosinophils increased (*P* < 0.05) quadratically with ZH supplementation compared with the CON group, with Z1 treatment having the highest values. Moreover, the results showed that Ossimi lambs had higher HB (*P* < 0.05) and neutrophils (*P* < 0.01), and lower basophils (*P* < 0.01) than Barki lambs. However, there was no significant effect of breed for HCT, RBC, platelets, MCH, MCHC, MCV, WBC, lymphocyte, monocyte, or eosinophil counts. Furthermore, no significant interactions among the breed × supplemented diet were observed (*P* > 0.05) for hematological parameters.

**Table 5:** Effect of breed type (B) and zilpaterol hydrochloride (ZH) treatment (T) and their interaction (B × T) on the hematological parameters of finishing lambs.

	Barki			Ossimi			*p* values			Contrast^b^	
Items	CON^a^	Z1	Z2	CON	Z1	Z2	B	T	B*T	L	Q
**HB**	11.6c ± 0.08	11.8^bc^ ± 0.16	12.0^a^^b^ ± 0.23	11.8^bc^ ± 0.05	12.1^a^ ± 0.14	12.4^a^ ± 0.25	*	**	ns	**	ns
**HCT, %**	38.6c ± 0.44	39.3^bc^ ± 0.49	40.7^a^ ± 0.79	38.8c ± 0.34	39.9^b^ ± 0.54	41.4^a^ ± 1.09	ns	**	ns	**	ns
**RBC, 10^6^/μL**	8.16c ± 0.65	8.58^b^ ± 0.70	9.01^a^ ± 0.81	8.40^bc^ ± 0.65	8.72^b^ ± 0.68	9.37^a^ ± 0.83	ns	**	ns	**	ns
**Platelets, 10^3^/μL**	394 ± 3.32	396 ± 3.01	395 ± 3.11	395 ± 3.21	399 ± 3.18	400 ± 3.22	ns	ns	ns	ns	ns
**MCH, pg**	12.0 ± 0.32	12.0 ± 0.36	12.0 ± 034	12.0 ± 0.52	12.0 ± 0.55	12.0 ± 0.58	ns	ns	ns	ns	ns
**MCHC, g/dL**	32.0 ± 1.33	32.0 ± 1.07	32.0 ± 1.12	32.0 ± 1.30	32.0 ± 1.37	32.0 ± 1.38	ns	ns	ns	ns	ns
**MCV, fL**	36.0 ± 1.82	36.0 ± 1.70	36.0 ± 1.79	36.0 ± 1.24	36.0 ± 1.63	36.0 ± 1.54	ns	ns	ns	ns	ns
**WBC, 10^3^/μL**	6.00 ± 0.29	6.00 ± 0.34	6.00 ± 0.38	5.99 ± 0.31	6.01 ± 0.37	5.99 ± 0.41	ns	ns	ns	ns	ns
**Neutrophils, %**	56.6^b^ ± 2.15	56.6^b^ ± 2.33	56.6^a^b ± 2.21	56.6^a^b ± 2.39	56.7^a^ ± 2.35	56.7^a^ ± 2.37	**	**	ns	**	ns
**Lymphocytes, %**	33.0 ± 1.12	33.0 ± 1.14	33.0 ± 1.10	33.0 ± 1.10	33.0 ± 1.18	33.0 ± 1.20	ns	ns	ns	ns	ns
**Monocytes, %**	5.99 ± 0.28	6.00 ± 0.33	5.99 ± 0.37	6.00 ± 0.30	6.00 ± 0.36	6.00 ± 0.40	ns	ns	ns	ns	ns
**Eosinophils, %**	3.99^b^ ± 0.21	4.01^a^ ± 0.17	4.00^ab^ ± 0.15	4.00^ab^ ± 0.12	4.01^a^ ± 0.18	4.00^ab^ ± 0.18	ns	*	ns	ns	*
**Basophils, %**	0.45^a^ ± 0.02	0.39^b^ ± 0.02	0.40^b^ ± 0.02	0.36^bc^ ± 0.02	0.34^c^ ± 0.01	0.35^c^ ± 0.01	**	ns	ns	ns	ns

*Note*: Means within the same row with different superscripts (^a–d^) are significantly different (*, *P* < 0.05; **, *P* < 0.01). Each value is expressed as mean ± standard error of the mean.

Abbreviations: HB, hemoglobin; HCT, hematocrit; RBC, red blood cells; MCH, mean corpuscular hemoglobin; MCHC, mean corpuscular hemoglobin concentration; MCV, mean corpuscular volume; WBC, white blood cells.

a Treatments: CON, control group; Z1, 0.10 mg/kg BW of ZH; Z2, 0.15 mg/kg BW of ZH.

b *p* values reported for the linear and quadratic contrasts of treatments.

### Blood biochemical composition

There were no significant influences of breed on blood biochemical parameters (Table 6; *P* > 0.05). Additionally, the breed type × supplemented diet interaction did not affect (*P* > 0.05) the blood biochemical parameters in this study. Dietary supplementation with ZH did not influence plasma BUN, creatinine, AST, ALT, or cortisol levels in this study. Plasma levels of TP, IGF1, T3, T4, and glucose increased linearly (*P* < 0.05) with dietary ZH supplementation. In contrast, plasma triglyceride and cholesterol levels were linearly lower (*P* < 0.01) with ZH supplementation, with CON lambs showing the highest levels.

**Table 6:** Effect of breed type (B) and zilpaterol hydrochloride (ZH) treatment (T) and their interaction (B × T) on the plasma biochemical parameters of finishing lambs.

	Barki			Ossimi			*p* values			Contrast^b^	
Items	CON^a^	Z1	Z2	CON	Z1	Z2	B	T	B × T	L	Q
**TP, g/dL**	5.89^b^ ± 0.25	5.90^b^ ± 0.35	6.48^a^ ± 0.43	5.88^b^ ± 0.25	5.99^b^ ± 0.15	6.56^a^ ± 0.35	ns	**	ns	**	**
**BUN, mg/dL**	17.6 ± 1.56	18.0 ± 0.67	17.6 ± 1.70	17.3 ± 0.74	18.2 ± 1.68	18.2 ± 0.75	ns	ns	ns	ns	ns
**Creatinine, mg/dL**	0.80 ± 0.04	0.79 ± 0.05	0.87 ± 0.04	0.82 ± 0.03	0.79 ± 0.04	0.81 ± 0.05	ns	ns	ns	ns	ns
**Glucose, mg/dL**	56.4^b^ ± 3.68	56.6^b^ ± 1.36	58.1^a^ ± 2.61	56.4^b^ ± 3.47	56.5^b^ ± 2.47	57.3^ab^ ± 1.45	ns	*	ns	*	ns
**Triglyceride, mg/dL**	35.0^a^ ± 0.62	33.0^a^ ± 0.46	29.5^b^ ± 1.27	35.1^a^ ± 0.85	33.0^a^ ± 0.47	29.6^b^ ± 1.24	ns	**	ns	**	ns
**Cholesterol, mg/dL**	104.4^a^ ± 0.40	97.8^b^ ± 1.45	96.2^b^ ± 1.65	103.7^a^ ± 0.32	97.6^b^ ± 1.44	96.7^b^ ± 1.70	ns	**	ns	**	*
**ALT, U/L**	50.6 ± 2.60	51.2 ± 3.68	52.1 ± 1.56	50.4 ± 4.54	51.1 ± 2.56	52.0 ± 2.76	ns	ns	ns	ns	ns
**AST, U/L**	121.1 ± 6.69	122.0 ± 10.6	121.9 ± 8.69	121.3 ± 12.8	121.8 ± 11.7	121.7 ± 10.7	ns	ns	ns	ns	ns
**Cortisol, µg/dL**	3.98 ± 0.08	3.98 ± 0.11	3.98 ± 0.08	3.96 ± 0.09	4.06 ± 0.06	3.85 ± 0.07	ns	ns	ns	ns	ns
**IGF1, ng/mL**	362.7c ± 10.6	367.1^b^ ± 6.59	369.2^b^ ± 8.62	364.0c ± 12.7	367.6^b^ ± 4.64	372.2^a^ ± 8.67	ns	**	ns	**	ns
**T3, ng/dL**	88.8^c^ ± 3.57	89.3^b^^c^ ± 2.88	91.4^a^ ± 5.12	88.1^c^ ± 3.56	90.8^a^^b^ ± 2.56	92.2^a^ ± 4.18	ns	**	ns	**	ns
**T4, ng/dL**	1.82c ± 0.04	2.12^b^ ± 0.06	2.37^a^ ± 0.10	1.89c ± 0.03	2.15^b^ ± 0.06	2.43^a^ ± 0.11	ns	**	ns	**	ns

*Note*: Means within the same row with different superscripts (^a–d^) are significantly different (*, *P* < 0.05; **, *P* < 0.01). Each value is expressed as mean ± standard error of the mean.

TP, total protein; BUN, blood urea nitrogen; ALT, alanine aminotransferase; AST, aspartate aminotransferase; IGF1, insulin-like growth factor 1; T3, triiodothyronine; T4, thyroxin.

a Treatments: CON, control group; Z1, 0.10 mg/kg BW of ZH; Z2, 0.15 mg/kg BW of ZH.

b *p* values reported for the linear and quadratic contrasts of treatments.

## Discussion

This study aimed to determine whether lambs from the Ossimi and Barki breeds would respond differently to ZH supplementation. The present study selected two distinct breeds, Ossimi and Barki, to implement the treatments because of their notable differences in body weight, meat quality, and fat content (Ashour et al. 2020; Fereig et al. 2023). Ossimi sheep exhibit greater body weight (Ashour et al. 2020), which accounts for the initial weight disparity between the two breeds, and they also have higher fat content than the Barki sheep (Ashour et al. 2020). However, the Barki breed is characterized by superior meat quality and low fat content (Abdel-Moneim 2009; Fereig et al. 2023).

### Growth performance and carcass traits

The inclusion of ZH in the diets of finishing lambs, particularly in the Ossimi and Barki breeds, linearly increased DMI, similar to the findings reported by Cayetano-De-Jesus et al. (2020). The DMI directly influences the gross or total energy available for growth (Guinguina et al. 2020). Therefore, we examined GE intake in finishing lambs of the Ossimi and Barki breeds and found significant increases, attributed to higher DMI. When ZH supplementation was applied, the enhanced DMI and GE intake likely contributed to better BW, ADG, and FCR. The anabolic action of ZH in finishing lambs is a multifaceted process that fundamentally alters the animal’s metabolism, favoring the accretion of lean muscle tissue over fat deposition (Lopez-Baca et al. 2019; Carrillo-Muro et al. 2023; Robles et al. 2024). This repartitioning effect is the primary driver of the observed improvements in growth performance and feed efficiency, by redirecting energy substrates away from fat deposition and towards muscle accretion. This also results in improved energy retention (Mersmann 1998). The primary mechanism by which ZH exerts its anabolic effects is by stimulating muscle hypertrophy through enhanced cAMP activity, which, in turn, inhibits proteolysis and lipogenesis while promoting protein synthesis (Mersmann 1998). Several studies have also reported greater ADG and feed efficiency when ZH is supplemented before slaughter in finishing lambs (Rojo-Rubio et al. 2018; Lopez-Baca et al. 2019; Carrillo-Muro et al. 2023). In contrast, other studies on sheep have reported no significant changes in these parameters (Vicente Pérez et al. 2022; Guerrero-Bárcena et al. 2023).

In addition, ZH has been reported to promote a shift in mass from non-carcass to carcass tissues, with greater substrate repartitioning in carcass tissues than in non-carcass tissues (Montgomery et al. 2009). In addition, Montgomery et al. (2009) observed a decrease in dry matter intake in ZH-treated cattle, resulting in reduced gut fill and increased dressing percentage. Carcass weight and yield improvements have been well-documented (López-Carlos et al. 2014; Avendaño-Reyes et al. 2016). However, variability in the responses to ZH supplementation exists, as some studies have reported no improvement in carcass weight and dressing percentage in lambs (Cayetano-De-Jesus et al. 2020; Vicente Pérez et al. 2022) or cattle (Fulton et al. 2024). This variability may be influenced by age, species, genetics, sex, diet, initial body weight, energy density, and dosage level (Aguilera-Soto et al. 2008; Estrada-Angulo et al. 2008).

In this study, dietary supplementation with ZH did not significantly affect carcass weight or dressing percentage. However, the interaction between breed and diet showed significant improvements in carcass weight and dressing percentage in Barki lambs fed ZH, echoing its well-documented role in finishing lambs in a feedlot setting (Carrillo-Muro et al. 2023; Robles et al. 2024). In the case of Ossimi lambs, the decrease in carcass weight and dressing percentage with ZH supplementation may suggest a lower sensitivity to ZH among heavier lambs. The recommended use of ZH is during the final 30 d of fattening (Yang and McElligott 1989), as prolonged use may decrease hot carcass weight, especially in animals with a limited capacity for weight gain (Strydom and Smith 2010). Overstimulation of β-adrenergic receptors by β-agonists causes receptor desensitization (Lohse et al. 1990), which downregulates adenylate cyclase catalytic activity and reduces cAMP synthesis (Pippig et al. 1993). The effects of ZH on whole cuts may vary owing to differences in muscle fiber types, with β-adrenergic agonists stimulating growth more in fast-fiber type muscles (Holmer et al. 2009). Therefore, the presence and quantity of type II muscle fibers can influence the extent of muscle hypertrophy induced by ZH, which stimulates the development of these fibers (Mersmann 1998; Vahedi et al. 2015). Thus, the possible variations in body composition, fat deposition patterns, and receptor distribution between the different breeds may be responsible for the differential effects of ZH between the two breeds in this study. For instance, Ossimi lambs typically possess higher initial fat reserves and distinct fat depot distributions, such as larger tail fat, whereas Barki lambs have leaner carcasses (Ashour et al. 2020). These physiological differences likely drive the breed-specific repartitioning effects of ZH, which tend to favor nutrient partitioning toward the carcass and muscle accretion rather than visceral organs or fat (Macías-Cruz et al. 2013). The significant breed × diet interaction in carcass composition compared to other parameters, such as growth performance or blood composition, likely results from ZH’s direct action on skeletal muscle and adipose tissues via β2-adrenergic receptor activation (Mersmann 1998; Johnson et al. 2014). This finding contrasts with reports in cattle, in which Fulton et al. (2024) noted a general lack of differential breed responses to ZH. Further research is needed to investigate breed-specific variations in β-adrenergic receptor expression and tissue-specific metabolic responses to ZH activation.

Dietary ZH mitigates the risk of excessive fat accumulation during the finishing phase by reducing or maintaining moderate carcass fat content, thereby increasing the proportion of carcasses within the preferred fat code range (Webb et al. 2024). The reduction in body fat with ZH treatment is attributed to increased lipolysis, decreased lipogenesis, and reduced proliferation of preadipocytes. β-Agonists activate β-adrenergic receptors, initiating the cAMP signaling pathway, which in turn activates rate-limiting enzymes in lipolysis and inactivates lipogenic enzymes involved in the synthesis and esterification of fatty acids within triacylglycerols (Johnson et al. 2014). This activation promotes the redistribution of energy substrates toward muscle tissue formation (Mersmann 1998). Several studies have reported reduced carcass subcutaneous fat in sheep supplemented with ZH (Vahedi et al. 2014; Guerrero-Bárcena et al. 2023; Robles et al. 2024), whereas others have observed reductions in internal fat depots such as kidney, pelvic, and heart fat (Estrada-Angulo et al. 2008). In contrast, other studies have reported no significant impact of ZH on lambs (Vicente Pérez et al. 2022), goats (López-Carlos et al. 2014), and cattle (Fulton et al. 2024). In our study, ZH supplementation reduced the chemical fat content and increased the protein content of the longissimus muscle in both breeds; however, breed-specific differences in fat deposition patterns were evident. Barki lambs with ZH supplementation showed low kidney and omental fat, but higher best rib fat, whereas Ossimi lambs with ZH showed low best rib fat, but higher kidney, omental, and tail fat. Previous studies have similarly highlighted breed-specific responses to β-adrenergic agonists. Huang et al. (2018) reported that reductions in adipose tissue mass can increase the deposition of dietary lipids in non-adipose tissues such as skeletal muscle and liver. Masoumi et al. (2021) demonstrated that ZH supplementation reduced subcutaneous fat mass but had no effect on fat-tail weight in fat-tailed lambs; moreover, the reduction in carcass fat was more pronounced in docked lambs than in intact lambs. Fat-tailed breeds channel a substantial proportion of dietary energy into tail fat deposition (Snyman et al. 2002), which reduces feed conversion efficiency owing to the high energy cost of this storage strategy (Masoumi et al. 2021). Consistent with these observations, dietary ZH did not significantly affect fat deposition in Iranian Lori-Bakhtiari fat-tailed lambs (Vahedi et al. 2014, 2015). The differential effects of ZH may be attributed to breed-specific fat deposition patterns with varying abundance, distribution, and adrenergic sensitivity of the β-agonist receptor subtypes in various tissues.

In this study, the interaction between ZH and the breeds showed significant enlargement of meat cuts in Barki lambs and a reduction in commertial cuts such as ribs, round, and shoulder, in Ossimi following ZH supplementation. The influence of ZH on carcass composition has been extensively studied, with varying results reported across different species. In lambs, ZH leads to increased carcass leanness and muscle proportion, including the breast, shoulder, loin, leg, and plain loin (Carrillo-Muro et al. 2023; Guerrero-Bárcena et al. 2023). Similarly, in goats, an evident increase in neck and leg perimeter has been observed (López-Carlos et al. 2014). In steers, ZH has been reported to increase subprime weights and cutting yields, particularly during the finishing period, with a more pronounced impact on the hindquarters (Johnson et al. 2014). However, not all studies have reported uniform improvements across all cuts. Some studies have indicated that ZH did not affect wholesale cuts in lambs, except for neck yield, which decreased with ZH supplementation (Robles et al. 2024). Additionally, a regionalized effect of ZH has been noted, with increased percentages of leg and hindquarter cuts and decreased forequarter cuts, such as the shoulder and neck (Rivera-Villegas et al. 2019). In contrast, other studies have not found an effect of ZH on the wholesale cut yields of lambs (Rojo-Rubio et al. 2018; Cayetano-De-Jesus et al. 2020), indicating potential variability in the response to ZH treatment.

In this study, we observed that ZH supplementation led to the enlargement of key muscle groups in Barki lambs, specifically the longissimus, supraspinatus, and semimembranosus muscles. This finding aligns with several studies indicating that ZH can enhance muscle growth in finishing lambs (Ortiz Rodea et al. 2016; Lopez-Baca et al. 2019; Guerrero-Bárcena et al. 2023). The enhanced longissimus muscle is primarily attributed to the higher ADG observed in the ZH-supplemented animals (Estrada-Angulo et al. 2016). This enhancement can be due to the anabolic effects of ZH, which stimulates protein synthesis and reduces protein degradation, thereby promoting muscle hypertrophy and reducing overall fat content (Yang and McElligott, 1989; Mersmann 1998). Furthermore, Ekpe et al. (2000) suggested that the enlargement of the longissimus muscle surface area in lambs administered a 15.9 mg/kg DM dosage of ZH may correlate with an increase in the diameter of type II muscle skeletal fibers. In contrast, the above-mentioned muscles were decreased in Ossimi lambs with ZH, which could be related to the lower carcass weight and dressing percentage with ZH supplementation compared to the CON group.

Moreover, dietary supplementation with ZH yielded contrasting effects on liver mass among Barki and Ossimi lambs in this study. Specifically, we observed a decrease in the liver mass of Ossimi lambs and an increase in that of Barki lambs with ZH. In lambs, ZH can reduce the weight of non-carcass components, such as the liver, skin, kidneys, spleen, rumen, and feet, according to Vicente Pérez et al. (2022). In addition, supplementation with ZH showed a more pronounced impact on carcass gain than on live weight gain in cattle (Strydom et al. 2009), leading to a proportional reduction in the weight of non-carcass components (Williams et al. 1987). Rivera-Villegas et al. (2019) proposed that the decrease in visceral organ mass could be a response to the increased energy demand associated with ZH supplementation. Furthermore, ZH acts as a repartitioning agent, redirecting nutrients from offal and adipose tissue to increase protein synthesis during the finishing phase, resulting in leaner carcasses (Vicente Pérez et al. 2022; Webb et al. 2024). However, other studies have demonstrated no significant effect of ZH on non-carcass components (Avendaño-Reyes et al. 2016). In addition, Castro-Pérez et al. (2024) showed no interaction between energy level and ZH on improvements in ADG and carcass weight or on the reduction of carcass fat in hairy lambs. The underlying cause of this inconsistency remains unclear; however, factors such as the dosage of ZH, genetic predispositions, age, and energy density of rations may influence the response to ZH supplementation.

### Meat quality

In the present study, we observed a linear increase in meat pH with ZH supplementation. The pH of meat is a pivotal attribute that influences its overall quality, including factors such as tenderness, water-holding capacity, and microbial growth (Dávila-Ramírez et al. 2017). While some studies have reported no significant effect of ZH on the pH of the longissimus muscle 24 h postmortem in lambs (Rojo-Rubio et al. 2018; Vicente Pérez et al. 2022), a considerable body of research has indicated an increase in meat pH in lambs fed ZH (Lopez-Baca et al. 2019; Cayetano-De-Jesus et al. 2020; Guerrero-Bárcena et al. 2023). The underlying mechanism for this increase involves a metabolic shift from aerobic to anaerobic metabolism induced by ZH, which is beneficial for protein accretion. However, this metabolic shift also leads to the rapid depletion of glycogen stores during slaughter. Consequently, reduced anaerobic glycolysis and lactic acid formation post-mortem could increase the muscle pH (Guerrero-Bárcena et al. 2023). However, the increased plasma glucose in Z2-treated lambs suggests that ZH-supplemented lambs do not have decreased available energy stores, which could affect animal performance (Buntyn et al. 2016).

Meat tenderness is a critical attribute of eating quality that directly influences consumer satisfaction and the likelihood of repurchasing red meat. While a substantial body of research has reported adverse effects of ZH on the tenderness and juiciness of cattle meat (Lopez-Baca et al. 2019; Fulton et al. 2024; Leyva-Medina et al. 2024), a handful of studies have found no detrimental impact on tenderness (O’Neill 2001). In the current study, we observed a linear reduction in Warner-Bratzler shear force in lambs treated with dietary ZH, indicating improved meat tenderness. This aligns with Hilton et al. (2009), who reported lower shear force in ZH-treated beef than in controls. The mechanism underlying this effect may involve ZH-induced alterations in ultimate muscle pH (Dávila et al. 2013; Lopez-Baca et al. 2019), which modulates both insoluble collagen and the activity of calpain–calpastatin proteolytic system (Wu et al. 2014). Wu et al. (2014) demonstrated that high-pH muscles exhibit accelerated myofibrillar protein degradation, whereas intermediate-pH muscles degrade proteins most slowly. Thus, the elevated ultimate pH observed in ZH-treated lambs could enhance post-mortem proteolysis, thereby improving tenderness. Additionally, ZH-mediated changes in the calpain–calpastatin system may influence muscle fiber hypertrophy and intramuscular fat catabolism (Strydom et al. 2009; Vahedi et al. 2015). Strydom et al. (2009) found that ZH reduced total collagen concentration in cattle Longissimus muscle, likely via a “dilution effect” from muscle hypertrophy (Kellermeier et al. 2009). ZH also decreases calpastatin activity (Strydom et al. 2009), leading to increased post-mortem proteolysis and more tender meat.

The WHC of meat is a fundamental quality characteristic that is intricately linked to the overall product quality. A reduced capacity to retain water can foreshadow a decrease in optimal quality, with significant losses potentially impacting a product’s visual appeal and consumer perception. In our study, lambs that received ZH exhibited a linearly higher WHC of meat, which contradicts the initial hypothesis that ZH treatment decreases WHC (Avendaño-Reyes et al. 2016). Interestingly, Lopez-Baca et al. (2019) reported that ZH-fed lambs tended to have increased WHC. The increased WHC could be due to increased meat pH in this study.

This study demonstrated that dietary ZH inclusion linearly increased meat protein content in both breeds, which is consistent with findings from previous sheep research (Guerrero-Bárcena et al. 2023). Dietary ZH has been observed to promote greater muscle nitrogen retention (Mersmann 1998), coupled with the redirection of resources from non-carcass components (Rojo-Rubio et al. 2018). In addition, the administration of ZH can cause peripheral vasodilation, increasing nutrient flow into muscles and providing more substrates for protein synthesis (Mersmann 2002). Supplementation with ZH increases the levels of myosin light chain, alpha-actin, and their mRNA transcripts, enhancing protein synthesis and muscle growth (Johnson et al. 2014). Furthermore, Giannetti et al. (2016) suggested that a decrease in lipolysis facilitates glycolysis, oxidation, and tricarboxylic acid cycle, potentially leading to increased protein synthesis. Thus, in this study, dietary supplementation with ZH linearly reduced the chemical fat composition of the meat. However, Neill et al. (2009) suggested that ZH did not significantly affect fat thickness or chemical fat content in three-rib cuts of cull cows. This discrepancy may highlight the potential variability in the responses to ZH supplementation across different species, breeds, or production conditions.

### Blood composition

ZH treatment is generally consistent across breeds for hematological and biochemical parameters. The levels of HCT, HB, RBC, and neutrophils increased linearly in lambs with the supplemention of ZH. However, Sieren (2016) found that ZH treatment increased HCT in heifers without significantly affecting HB, RBC, WBC, or neutrophils. Conversely, ZH supplementation decreased the HB and HCT values in male goats during the finishing phase (Hatefi et al. 2017). It did not affect HCT, HB, or RBC in fattening hair sheep (Vicente Pérez et al. 2020). In horses, ZH administration resulted in insignificant changes in HCT, HB, WBC, lymphocytes, and monocytes (Wagner et al. 2008). However, microscopic examination revealed acanthocytes and neutrophils with toxic changes associated with ZH administration (Wagner et al. 2008). In this study, the increase in neutrophils and eosinophils with ZH treatment suggests a potential immune response or stress, consistent with the findings in cattle, where adrenergic agonists such as ZH demonstrated immune activation (Howell et al. 2024). This immune response could be due to ZH’s β-agonist properties of ZH, which may activate the immune system and increase neutrophils and eosinophils as part of the body’s defense. Meanwhile, the sirtuin and IL-7 signaling pathways associated with stress and immune pathways were downregulated in beef cattle subjected to heat stress following the administration of ZH (Reith et al. 2022).

β-agonists, such as ZH, promote muscle growth and protein synthesis, decrease protein catabolism, and increase tissue nitrogen deposition (Mersmann 1998). This could explain the linear increase in the TP levels observed in this study. There was a significant linear effect on IGF1 levels, with the Z2 group showing the highest levels. IGF1 is an anabolic hormone that promotes growth and cell proliferation, and its increase with ZH treatment may be related to the growth-promoting effects of β-agonists (Parr et al. 2014). The highest glucose levels were observed in the Z2 group in both breeds. Similarly, Eisemann et al. (1988) performed a study in which steers were fed 8 mg/d of clenbuterol daily for 9 d. The authors observed an initial increase in the glucose concentration. The authors suggested an initial increase in glucose entry rate due to increased liver gluconeogenesis or glycogenolysis (Eisemann et al. 1988). In addition, the levels of both T3 and T4 increased linearly with ZH treatment, which may be related to increased metabolic rate, muscle growth, and fat loss by affecting the endocrine system (Hatefi et al. 2017).

Moreover, the plasma triglyceride and cholesterol levels reduced linearly with ZH treatment. This is consistent with the lipolytic effects of β-agonists, which may support the oxidation of fatty acids and reduce the number of fatty acids available for re-esterification into triglycerides. This aligns with Giannetti et al. (2016), who indicated that during lipolysis in sheep, triglycerides are broken down into glycerol and free fatty acids and facilitate glycolysis, oxidation, and the tricarboxylic acid cycle. In addition, the increase in thyroid hormone levels in this study with ZH treatment could reduce blood cholesterol levels. It has been reported that the thyroid hormone receptor-β selective agonists could affect cholesterol metabolism by upregulating the expression of low-density lipoprotein receptors in the liver (Lin et al. 2012). Further research is necessary to understand how ZH affects liver function and develop strategies to mitigate its adverse effects. A limitation of this study is that samples were collected only at day 30 without baseline measurements. Future studies should incorporate baseline sampling to strengthen causal inference.

## Conclusion

In summary, Ossimi lambs showed superior growth, carcass traits, dressing percentage, and hematological values, whereas Barki lambs had a higher meat protein content. ZH supplementation was associated with improved growth performance, meat quality (protein, WHC, pH), hematological indices (HCT, HB, RBC), immune markers (neutrophils and eosinophils), and anabolic hormone concentrations (IGF1, T3, and T4) and decreased plasma triglycerides and cholesterol, meat fat, and Warner-Bratzler shear force. Notably, Barki lambs responded more favorably to ZH in terms of carcass traits, suggesting breed-specific responses.

## Data Availability

The datasets generated during and/or analyzed during the current study are available from the corresponding author on reasonable request.

## Abbreviations

ADG: Average daily gain
ALT: alanine aminotransferase
ANOVA: analysis of variance
AST: aspartate aminotransferase
BUN: blood urea nitrogen
BW: body weight
CBC: complete blood count
FMD: foot and mouth disease
HB: hemoglobin
HCT: hematocrit
IGF1: insulin-like growth factor 1
MCH: mean corpuscular hemoglobin
MCHC: mean corpuscular hemoglobin concentration
MCV: mean corpuscular volume
RBC: red blood cell
T3: triiodothyronine
T4: thyroxin
TG: total gain
TP: total protein
WBC: white blood cell count
WBS: Warner-Bratzler Shear Force
WHC: water holding capacity
ZH: zipatrol hydrochloride
